# *IFITM3* Rs12252-C Variant Increases Potential Risk for Severe Influenza Virus Infection in Chinese Population

**DOI:** 10.3389/fcimb.2017.00294

**Published:** 2017-06-30

**Authors:** Yang Pan, Peng Yang, Tao Dong, Yi Zhang, Weixian Shi, Xiaomin Peng, Shujuan Cui, Daitao Zhang, Guilan Lu, Yimeng Liu, Shuangsheng Wu, Quanyi Wang

**Affiliations:** ^1^Institute for Infectious Disease and Endemic Disease Control, Beijing Center for Disease Prevention and ControlBeijing, China; ^2^Research Centre for Preventive Medicine of BeijingBeijing, China; ^3^Capital Medical University School of Public HealthBeijing, China; ^4^MRC Human Immunology Unit, Weather all Institute of Molecular Medicine, University of OxfordOxford, United Kingdom

**Keywords:** influenza, interferon inducible transmembrane 3, single nucleotide polymorphism, severe acute respiratory infection, genetic susceptibility

## Abstract

**Background:** Interferon Inducible Transmembrane 3 (IFITM3) is a key factor in interferon pathway and it involves host's immune response against multiple viruses. *IFITM3* rs12252-C was associated with severe influenza virus infection in several studies, however whether this association is universal to all types of influenza virus or diverse ethnic populations remain controversial.

**Method:** A case-control genetic association study was performed from September 2013 to April 2014 and September 2014 to April 2015. All samples were tested for influenza using RT-PCR, and genotyped by High Resolution Melting assay.

**Results:** A total of 65 healthy people, 165 mild influenza-like illness (ILI) cases and 315 severe acute respiratory infection (SARI) cases were enrolled in this study. The frequency of CC genotype was much higher in SARI cases with IVI than that in ILI cases with IVI (61.59 vs. 27.16%), leading a 4.67-fold greater risk for severe IVI than other two genotypes. Moreover, the risk of *IFITM3* rs12252-C variant for severe IVI was specific for both influenza A and influenza B.

**Conclusion:** *IFITM3* rs12252 CC genotype was associated with severity rather than susceptibility of IVI in Chinese population, and this strong effect was observed in all subtypes of seasonal influenza infection.

## Introduction

Influenza has been a constant global health concern since the pandemic of 1918. Currently, influenza A (H1N1 09pdm), A(H3N2), B(Victoria) and B(Yamagata) are the most common subtypes/lineages in influenza virus infection (IVI). Most people with IVI show mild influenza-like illness (ILI) symptom, including fever, nasal discharge, sore throat, cough, headache, etc., and will recover in several days (Hayward et al., 2014). However, some IVI cases may develop severe infection and a low percent of them even need intensive care unit (ICU) admission or die of persistent infection (Dunning et al., 2014; Thomas, 2014). In the United Kingdom, the mean seasonal hospitalizations attributed to IVI were up to 48/100,000, and a quarter of them died of respiratory disease due to IVI (Matias et al., 2016). Thus, identifying the potential risk factor for severe IVI is crucial to formulating effective preventing and managing strategies.

As interferon inducible transmembrane 3 (IFITM3) is a type 1 interferon inducible transmebern protein, it can restricte many RNA virus entry to the cells including influenza, SARS, ebola, hepatitis C virus (Brass et al., 2009). It is also known that several influenza virus strain in particular of avian origin have evolved to escape IFITM3 restriction (Sun et al., 2016). A single nucleic variation within IFITM3 has been shown to be associated with sever influenza outcome (Everitt et al., 2012; Zhang et al., 2013). Previous studies predicted that rs12252-C might produce an alternate spliced transcript that encodes an aberrant truncated protein (Δ21 IFITM3), which reduces the cellular resistance to influenza viruses by blocking early stage of viral replication (Everitt et al., 2012; Compton et al., 2016). However, the expression of Δ21 IFITM3 variant at mRNA or protein level has not been verified. Everitt AR et al. showed the association between *IFITM3* rs12252-C variant associated Δ21 IFITM3 and severe infection *in vitro*, while it was controversial whether this association also existed in human populations. The association was observed in studies performed in England and China (Everitt et al., 2012; Zhang et al., 2013; Wang et al., 2014). However, similar studies from Portuguese, Spain and the United Kingdom showed negative results (Mills et al., 2014; Gaio et al., 2016; Lopez-Rodriguez et al., 2016).

It is noteworthy that the allele frequency of *IFITM3* rs12252-C and people carrying CC genotype in Europe is rare (0.3%), while up to 25% of Han Chinese population carry CC genotype, which may greatly increase the power of analysis (Abecasis et al., 2012). It is therefore not so surprising that three studies did not observe the association because the population used for the analysis was based on the Caucasians where the C allele is extremely rare in their cohorts. Therefore, in this study, we randomly enrolled 156 mild ILI cases and 315 severe acute respiratory infection (SARI) cases, investigating the possible association between rs12252-C allele and severe infection from seasonal influenza infection including H1N1, H3N2, and influenza B viral strains.

## Materials and methods

### Study design and subjects

A case-control genetic association study was employed to compare the frequency of *IFITM3* rs12252 variant C between healthy population, ILI cases and SARI cases. Ethical approval was obtained from the institutional review board and human research ethics committee of the Beijing Center for Disease Prevention and Control (BCDC). Oral agreement was obtained when the case was enrolled in this study, and written informed consent as a part of questionnaires were completed by each participant after the sampling.

The surveillance for SARI included 11 inpatient departments in local hospitals located in urban and suburban districts of Beijing area. The enrolment criteria for SARI cases included: (a) inpatients with a temperature >38°C and cough; (b) onset of clinical symptoms within 10 days (World Health Organization, 2014). We also enrolled ILI cases as mild controls in this study. The sentinel sites included 11 outpatient departments in local hospitals located in urban and suburban districts of Beijing area. The enrolment criteria for ILI cases included: (a) outpatients seeking medical care at the designated sentinel hospitals for ILI, defined as an SARI with a temperature >38°C and cough or sore throat; (b) onset of ILI clinical symptoms within 10 days; and (c) no anti-viral treatment applied (World Health Organization, 2014). The subjects of this study were randomly selected in patients with SARI and ILI from September 2013 to April 2014 (2013-14 influenza season) and September 2014 to April 2015 (2014-15 influenza season). Healthy people who did health examination in Beijing Hospital from September 2014 to April 2015 were also enrolled in this study.

Throat swabs were collected from the enrolled patients at the day of the visit. Specimens were stored in 3 mL of virus transport medium at 4°C and tested within 24 h. Meanwhile, information questionnaires, including the demographic information, vaccine inoculation, etc. were completed by participating physicians at the same time. For healthy controls, anticoagulant blood was collected during the health examination and kept at −20°C until testing.

### Influenza A/B virus testing

All specimens collected from ILI and SARI cases were typed and subtyped for influenza A/B virus by real-time reverse transcription-polymerase chain reaction (RT-PCR) according to the protocol of the Chinese National Influenza Centre (CNIC) (Chinese National Influenza Center, 2012). Briefly, viral RNA was extracted from the samples using QIAmp Viral Mini Kit (Qiagen, Hilden, Germany) following the manufacture's instruction. Then real-time RT-PCR was performed to identify the influenza A/B viral RNA using AgPath ID One-step RT-PCR Kit (Applied Biosystems, California, USA) and 7500 real-time PCR system (Applied Biosystems).

### DNA extraction and genotyping

Genomic DNA was extracted from 300 μl of throat swab samples (for ILI cases and SARI cases) or 100 μl of anticoagulant blood(for healthy controls) using QIAamp DNA Micro kit (Qiagen) following the manufacturer's instruction. Extracted DNA integrity was checked by NanoDrop ND-1000 (NanoDrop Technologies, Wilmington, U.S.A.), then kept at −40°C before testing. Genotype was achieved using Type-it HRM PCR Kit (Qiagen) with LightCycler 480 System (Roche, Zurich, Swiss) following the manufacturer's instruction. The High Resolution Melting (HRM) was carried out with the pair of primers 5′-GGAAACTGTTGAGAAACCGAA-3′ and 5′- CATACGCACCTTCACGGAGT-3′ (Zhang et al., 2013). In order to confirm the results of genotypes, 10% of the samples were randomly selected and sequenced. PCR was performed using AmpliTaq Gold 360 Master Mix Kit (Applied Biosystems) and the primers as described above. Then the PCR products were sequenced on 3130xl genetic analyzer (Applied Biosystems). The sequencing results were analyzed by Chromas 2.6 software (Technelysium, Brisbane, Australia).

### Statistical analysis

Allelic and genotypes of individuals were calculated by direct counting and were tested for Hardy-Weinberg equilibrium (HWE) using online software (https://ihg.gsf.de/cgi-bin/hw/hwa1.pl) (Rohlfs and Weir, 2008). Data was analyzed using SPSS 20.0 (SataCorp, College Station, USA). Difference between groups were evaluated using Pearson's χ2, Fisher's exact test or logistic regression, and odds ratio (ORs) together with 95% confidence intervals (CI) were estimated. Meanwhile, subjects were stratified by age as 0–6, 7–18, 19–40, 41–65, and older than 65. Then clinical characteristics (stratified age, gender, and vaccination) between different groups were adjusted using logistic regression models, to calculate the adjusted ORs (aORs) with corresponding 95% CI. In addition, a test-negative design was adopted to estimate the influenza vaccine effectiveness (VE) against SARI using logistic regression models. A *p* < 0.05 was considered statistically significant.

## Results

### Clinical characteristics of enrolled subjects

A total of 65 healthy people, 165 ILI cases (including 84 non-IVI cases and 81 IVI cases) and 315 SARI cases (including 151 non-IVI cases and 164 IVI cases) in Beijing area were surveyed, tested and analyzed in this study (Table 1). No significant differences were observed regarding the gender distributions between these three group (*p* = 0.923). However, the age of SARI cases was higher than that of ILI cases (*p* < 0.05).

**Table 1:** Clinical characteristics of study subjects.

**Characteristics**	**Controls (*n* = 65)**	**ILIs (*n* = 165)**	**SARIs (*n* = 315)**
Age (years)^a^	43.25 ± 11.52	40.51 ± 24.97	47.45 ± 29.53
Gender (female/male)	35/30	84/81	163/152
Vaccination (%)	-	17 (10.30%)	40 (12.70%)

*ILI, influenza-like illness, SARI, Severe Acute Respiratory Infection , -, undetermined*.

a *Data is shown as mean ± SD*.

### *IFITM3* rs12252 CC genotype was associated to severity rather than susceptibility of IVI

**Figure 1:**
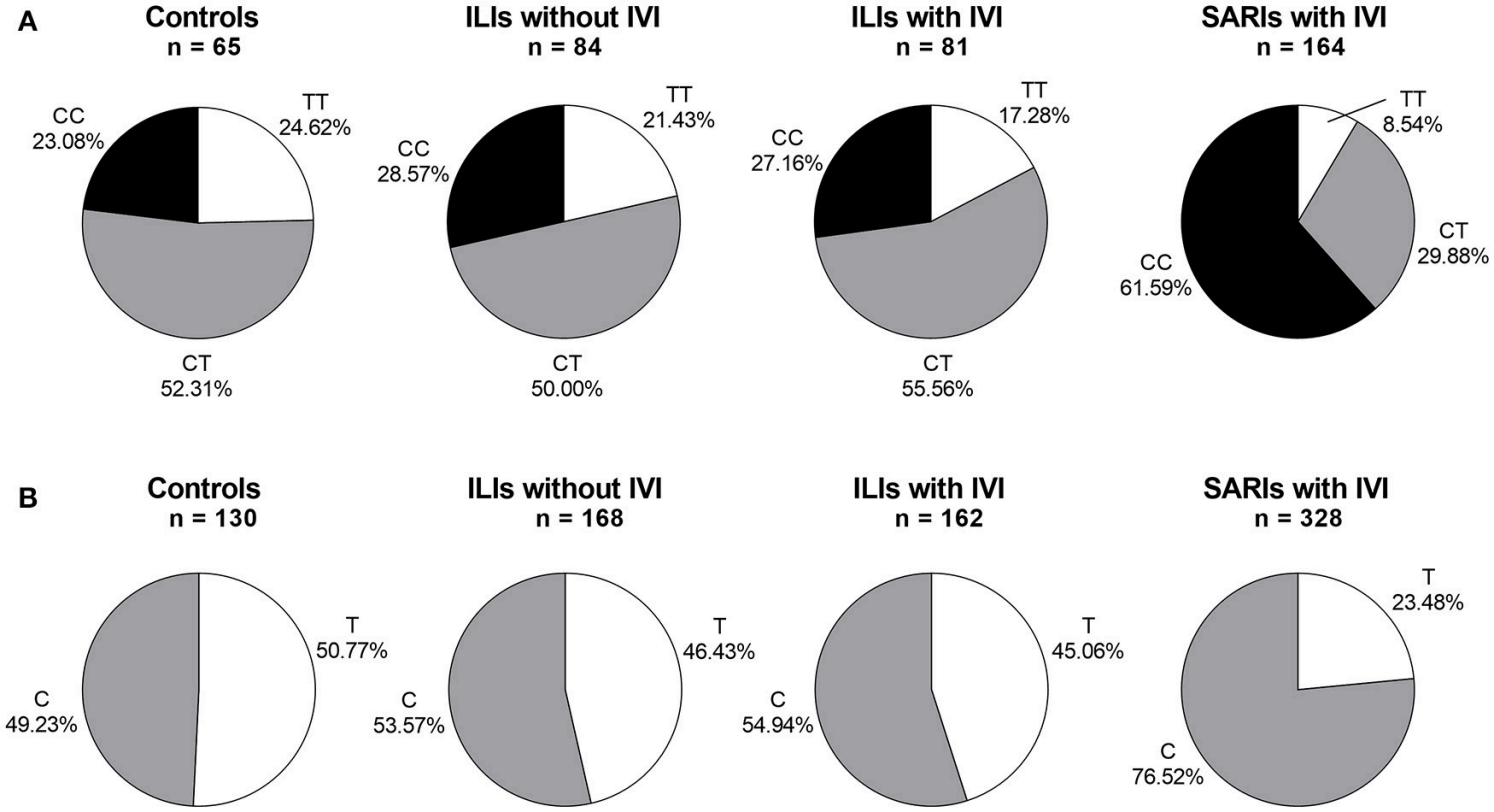
*IFITM* rs12252 genotypic and allelic frequencies in controls, ILI cases and SARI cases. **(A)** *IFITM* rs12252 genotypic frequencies in controls, ILI cases and SARI cases. **(B)** *IFITM* rs12252 allelic frequencies in controls, ILI cases and SARI cases. ILI, influenza-like illness, SARI, Severe Acute Respiratory Infection, IVI, influenza virus infection.

The *IFITM3* rs12252 genotype was in Hardy-Weinberg equilibrium in all tested groups except for SARI cases with IVI (*p* < 0.05). The genotyping for *IFITM3* rs12252 showed that 23.08% of healthy controls and 27.88% of ILI cases carried CC genotype, both of which was significantly lower than that of SARI cases (55.87%, *p* < 0.001, and *p* < 0.001, respectively). More specifically, the SARI cases with IVI owned a CC genotype frequency of 61.59%, while the ILI cases with IVI had a much lower CC genotype frequency of 27.16% (*p* < 0.001, Figure 1). However, no differences on genotypic distribution were observed between ILI cases with IVI and ILI cases without IVI (*p* = 0.728).

### 4.67-fold risk for severe infection in IFITM3 rs12252-C variants was calculated by recessive model

To further assess the potential risk of *IFITM3* rs12252-C variant in severe infection cases, we performed a stratified analysis using additive model, dominant model, recessive model and allelic/multiplicative model. The association of genotypic distribution and severe IVI was demonstrated by all four models(*p* < 0.001, *p* < 0.05, *p* < 0.001, and *p* < 0.001, respectively, Table 2) Among them, recessive model showed the highest potential risk, with a crude OR of 4.299 (95% CI: 2.402-7.694) and an adjusted OR of 4.673 (95% CI: 2.558-8.537).

**Table 2:** Risk assessment for rs12252 genotype using different models.

**Genetic model**	**ILIs with IVI (*n* = 81)**	**SARIs with IVI (*n* = 164)**	**OR (95% CI)**	***p*-value**	**aOR (95% CI)**^a^	***p*-value**^a^
**ADDITIVE**						
CC (%)	22 (27.16%)	101 (61.59%)	2.543 (1.690–3.838)	<0.001	2.638 (1.735–4.009)	<0.001
CT (%)	45 (55.56%)	49 (29.88%)				
TT (%)	14 (17.28%)	14 (8.54%)				
**DOMINANT**						
CC+CT (%)	67 (82.72%)	150 (91.46%)	2.239 (1.011–4.957)	<0.05	2.242 (1.004–5.006)	<0.05
TT (%)	14 (17.28%)	14 (8.54%)				
**RECESSIVE**						
CC (%)	22 (27.16%)	101 (61.59%)	4.299 (2.402–7.694)	<0.001	4.673 (2.558–8.537)	<0.001
CT+TT (%)	59 (72.84%)	63 (38.41%)				
**ALLELIC/MULTIPLICATIVE**						
C (%)	89 (54.94%)	251 (76.52%)	2.674 (1.790–3.994)	<0.001	–	–
T (%)	73 (45.06%)	77 (23.48%)				

*ILI, influenza-like illness, SARI, Severe Acute Respiratory Infection, IVI, influenza virus infection, CI, confidence interval, OR, odds ratio, aOR, adjusted odds ratio, -, undetermined*.

a *adjusted for age, gender and vaccination*.

### The risk of rs12252-C variant for severe IVI was specific for both influenza A (H1N1 and H3N2) and influenza B

**Figure 2:**
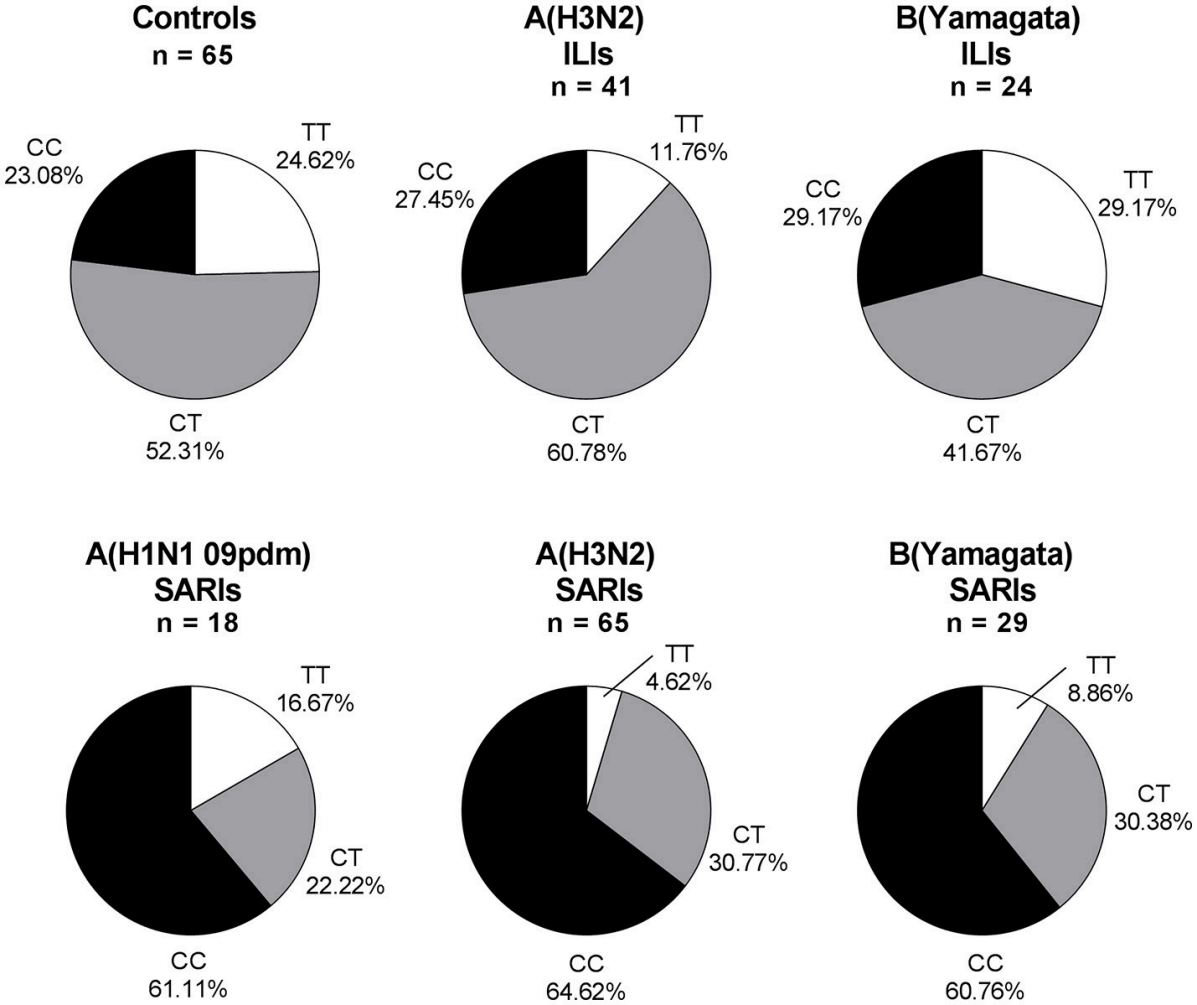
*IFITM* rs12252 genotypic frequencies in IVI cases with different viral type. ILI cases and SARI cases. ILI, influenza-like illness, SARI, Severe Acute Respiratory Infection, IVI, influenza virus infection. Five influenza A(H1N1 09pdm) virus-infected ILIs (including one CC genotype and four CT genotype), one influenza B(Victoria) virus-infected ILI (TT genotype) and two influenza B(Victoria) virus-infected SARIs (one TT genotype and one CT genotype) were not shown.

Overall, 245 ILI and SARI cases were positive in influenza A/B testing, including 23 A(H1N1 09pdm) viruses, 106 A(H3N2) viruses, 3 B(Victoria) viruses and 113 B(Yamagata) viruses. Regarding the different viral subtype or lineage of IVI, we found that *IFITM3* rs12252 CC genotype was associated with severe infection in total influenza A, influenza A (H3N2), total influenza B and B (Yamagata) virus-infected cases (*p* < 0.001, *p* < 0.001, *p* < 0.01, and *p* < 0.01, with crude OR of 4.83, 4.83, 3.74, and 3.76, respectively). However, the association was not clear in B (Victoria) virus-infected cases, possibly due to the limited sample size (Figure 2). There were only 5 pdmH1N1 infected cases and none of these 5 cases were TT genotype so we did not include them as a separate group.

## Discussion

The association between *IFITM3* rs12252-C variant and IVI was first reported by Everitt et al. (2012). Since then, a series of studies were aimed to verify this conclusion (Everitt et al., 2012; Zhang et al., 2013; Mills et al., 2014; Wang et al., 2014; Gaio et al., 2016; Lopez-Rodriguez et al., 2016). However, due to the limited sample size and the racial disparity, our knowledge about this issue is far from clear. In a more recent study, Lopez-Rodriguez et al. enrolled the largest number of participants of 118 Spanish Caucasian patients. However, because the CC genotype in North Americans and Europeans is rather low, only one patient with CC genotype was found in mild IVI group of 58 patients, whereas no such a genotype was observed in severe IVI group of 60 hospitalized patients (Lopez-Rodriguez et al., 2016). Likewise, Mills et al found two individuals with CC genotype in a group of 259 patients with mild IVI and four such individuals in a group of 2623 controls, but no individual with CC genotype was identified in 34 cases with IVI requiring ICU admission (Mills et al., 2014). Although these data suggested a potential role of rs12252-C variant in susceptibility to mild IVI rather than severe IVI in Europeans, it still needs to be verified owing to the extremely low frequency of CC genotype in these areas. Hence in the present study, we designed a large-scale investigation to identify the rs12252-C variant in Chinese population, which possess high frequency of CC genotype in this site. This investigation was carried out based on the surveillance for SARI cases. A regional surveillance for SARI has been established by BCDC since July 2014. Based on the surveillance data, more than 50% of SARI cases were of IVI at the peak of influenza season (data was not shown), and other respiratory viruses were comparatively uncommon in samples collected during the influenza season. Thus, the SARI cases were treated as population with severe infection and compared with mild ILI cases to evaluate the effect of rs12252-C variant.

As one of the most important findings in this study, the association between *IFITM3* rs12252-C variant and the severe influenza infection is observed. Our data showed that the CC genotype of *IFITM3* rs12252 was more frequent in severe infection cases rather than in mild cases. Meanwhile, this variant did not lead to an increase risk for IVI. These results concur with those of Zhang et al. (2013), who also found an association with severe but not mild IVI. Interestingly, all the studies performed in China confirmed the association between rs12252-C variant and the severe IVI. However, no such investigations carried out in Europe gave a similar result. The distinct allele distribution of rs12252-C variant in different population should be part of the reason. Therefore, more studies with other large populations are needed to further define this genetic association. More importantly, possible linkage disequilibrium (LD) with other variants at IFITM3 or nearby genes, which associate with IVI, may also contribute to this difference. It should not be ignored in the following studies.

The association between rs12252-C variant and IVI was based on the central role of IFITM3 in limiting the entry and replication of influenza virus (Smith et al., 2014; Williams et al., 2014). This effect has been confirmed *in vitro* and *in vivo*, and not restricted by the type or subtype of influenza virus (Bailey et al., 2012; Perreira et al., 2013; Smith et al., 2014). In this study, the risk of *IFITM3* rs12252-C variant for severe IVI was observed in seasonal A(H3N2) and B(Yamagata) IVI. Combining with previous studies, it is clearly that this association has been observed in Chinese population with pandemic influenza (H1N1 09pdm), seasonal influenza (H3N2 and influenza B) and avian influenza (H7N9).

In conclusion, we showed here that the *IFITM3* rs12252-C variant acts as a risk for severe IVI, but not for the susceptibility to IVI. Moreover, this risk of exists in both seasonal influenza A (H1N1 and H3N2) and influenza B infection. Therefore, the potential value of *IFITM3* rs12252-C variant, treating as a diagnostic biomarker for severe IVI, is meaningful and noteworthy.

## Ethics statement

This study was carried out in accordance with the recommendations of institutional review board and human research ethics committee of the BCDC with written informed consent from all subjects. All subjects gave written informed consent in accordance with the Declaration of Helsinki. The protocol was approved by the institutional review board and human research ethics committee of the BCDC.

## Author contributions

YP and PY: Study design, data analysis, data interpretation, writing of the manuscript; TD: Data analysis, data interpretation, revising the manuscript; YZ, DZ, and SW: Study implementation, data collection, data reduction; WS, XP, SC, and GL: Gene testing, sequencing, data collection; YL: Influenza testing; QW: Initiating the study, writing of the manuscript, revising the manuscript, final approval of the version to be published.

### Conflict of interest statement

The authors declare that the research was conducted in the absence of any commercial or financial relationships that could be construed as a potential conflict of interest.
